# Inhibition of SHP2 in basal-like and triple-negative breast cells induces basal-to-luminal transition, hormone dependency, and sensitivity to anti-hormone treatment

**DOI:** 10.1186/s12885-015-1131-2

**Published:** 2015-03-08

**Authors:** Hua Zhao, Yehenew M Agazie

**Affiliations:** Department of Biochemistry and The Marry Babb Randolph Cancer Center School of Medicine, West Virginia University, Morgantown, WV 26506 USA

**Keywords:** SHP2, ERα, Breast cancer, Invasiveness, Basal-to-luminal transition, Tamoxifen

## Abstract

**Background:**

The Src homology phosphotyrosyl phosphatase 2 (SHP2) is a positive effector of cell growth and survival signaling as well transformation induced by multiple tyrosine kinase oncogenes. Since the basal-like and triple-negative breast cancer (BTBC) is characterized by dysregulation of multiple tyrosine kinase oncogenes, we wanted to determine the importance of SHP2 in BTBC cell lines.

**Methods:**

Short hairpin RNA-based and dominant-negative expression-based SHP2 inhibition techniques were used to interrogate the functional importance of SHP2 in BTBC cell biology. In addition, cell viability and proliferation assays were used to determine hormone dependency for growth and sensitivity to anti-estrogen treatment.

**Results:**

We show that inhibition of SHP2 in BTBC cells induces luminal-like epithelial morphology while suppressing the mesenchymal and invasive property. We have termed this process as basal-to-luminal transition (BLT). The occurrence of BLT was confirmed by the loss of the basal marker alpha smooth muscle actin and the acquisition of the luminal marker cytokeratin 18 (CK18) expression. Furthermore, the occurrence of BLT led to estrogen receptor alpha (ERα) expression, hormone dependency, and sensitivity to tamoxifen treatment.

**Conclusions:**

Our data show that inhibition of SHP2 induces BLT, ERα expression, dependency on estrogen for growth, and sensitivity to anti-hormone therapy. Therefore, inhibition of SHP2 may provide a therapeutic benefit in basal-like and triple-negative breast cancer.

**Electronic supplementary material:**

The online version of this article (doi:10.1186/s12885-015-1131-2) contains supplementary material, which is available to authorized users.

## Background

The recent decline in breast cancer death rate is attributed, at least in part, to availability of targeted therapies such as Herceptin against HER2-positive and tamoxifen against estrogen receptor-positive breast cancers [1]. Unfortunately, no such treatment options exist for the basal-like and/or triple-negative breast cancer (BTBC). As a result, BTBC causes disproportionately high mortalities in women [2], mainly in African-American women and in younger women of all ethnicities. The term basal-like was derived from the expression profile of basal cytokeratins (CK5/6, CK14 and CK17) by BTBC tumors, proteins expressed by the basal cells of the normal breast, the myoepithelial cells [1,3]. But, recent reports suggest that BTBC may also originate from pluripotent luminal cells [4]. Another characteristic feature of BTBC tumors is the elevated expression of the epidermal growth factor receptor (EGFR) and multiple other receptor tyrosine kinases (RTKs), including the MET, the FGFR, and the IGF-1R [5-8].

The Src homology phosphotyrosyl phosphatase 2 (SHP2) is an essential transducer of mitogenic and cell survival signaling downstream of multiple RTKs, including those dysregulated in BTBC [9-11]. In addition, SHP2 is important for cell transformation induced by oncogenic RTKs and v-Src [12-15]. It was thus reasonable to determine the importance of SHP2 in BTBC cell lines in which multiple RTKs are known to be dysregulated. SHP2 is composed of two Src homology 2 domains in the N-terminal and a PTP domain in the C-terminal regions [16,17]. The SH2 domains allow interaction with phosphotyrosine while the PTP domain dephosphorylates target substrates. In a resting state or in the absence of tyrosine kinase signaling, SHP2 assumes a closed inactive confirmation due to intramolecular interaction between the N-terminal SH2 and the PTP domains. The binding of the SH2 domains to phosphotyrosine disrupts the intramolecular interaction, leading to an open and active confirmation. Hence, increased tyrosine kinase signaling induced by dysregulated RTKs in BTBC can lead to increased SHP2 activity and augmented downstream signaling. In this report, we show that inhibition of SHP2 in BTBC cells reverses the mesenchymal phenotype, abolishes invasiveness, induces basal-to-luminal transition (BLT), and confers hormone dependency and sensitivity to anti-hormone (tamoxifen) treatment.

## Methods

### Cells, cell culture and reagents

The MDA-MB231 and the MDA-MB468 breast cancer cell lines and the MCF-10A cells were purchased from ATCC. These cells were grown as described previously [18,19]. The anti-β-actin monoclonal antibody (A5441) was from Sigma-Aldrich, the anti-Snail antibody (SN9H2) was from Cell Signaling, the anti-EGFR antibody (610017) was from BD Biosciences, the anti CK18 antibody (M7010) was from DAKO, the anti-smooth muscle actin (MA1-26017) and the anti-estrogen receptor alpha (MA1-310) antibodies were from Thermo Scientific, and the anti-MMP2 (MAB3308) and the anti-MMP9 (AB13458) antibodies were from Millipore. The anti-SHP2 (SC-7384), the anti-vimentin (SC-32322), the anti-progesterone receptor (SC-538), and the anti-fibronectin (SC-18825) antibodies were from Santa Cruz Biotechnology. Anti-mouse and anti-rabbit secondary antibodies conjugated with horseradish peroxidase were purchased from Jackson Immuno-Research Laboratories.

### Inhibition of SHP2 by shRNA and by dominant-negative expression

Two independent shRNA sequences (double-stranded deoxyoligonucleotides) previously shown to be specific for SHP2 [18,20,21] were used for silencing of SHP2 in the MDA-MB231 and MDA-MB468 cells. A short hairpin RNA against luciferase was used as a control as also described previously [18].

### Preparation of cell lysates and immunostaining analyses

Cell lysates were prepared in a buffer containing 20 mM Tris-HCl, pH7.2, 150 mM NaCl, 50 mM NaF, 1 mM EDTA, 10% glycerol, 1% triton-X-100, 1 mM sodium orthovanadate and a protease inhibitor cocktail. For total cell lysate analysis, proteins were separated using a standard polyacrylamide gel electrophoresis, transferred onto nitrocellulose membranes, blocked in 3% bovine serum albumin, stained with primary antibodies overnight at 4°C, washed three times with TBST (Tris-buffered saline containing 0.1% Tween-20) and incubated with secondary antibodies for 1 hour at room temperature. Finally, stained bands were detected by the chemiluminescence method.

### 2D monolayer wounding and 3D laminin-rich basement membrane (LRBM) culture

For monolayer wounding assay, cells were grown in DMEM containing 10% FBS and scratched with a pipet tip to create a gap. The ability of the cells to migrate and fill the space was monitored under a microscope. Pictures were collected under an IX-71 Olympus microscope using the 5 × objective immediately after wounding and every 12 hours thereafter.

For 3D invasion assay, a modified version of the laminin-rich basement membrane (LRBM) culture, sometimes referred to as 3D matrigel assay, was used. The basic protocol has been previously reported [22]. Four-well chamber slides (Falcon) were overlaid with 80 μl of growth factor reduced LRBM medium (BD Biosciences) containing 10 μg/ml DQ collagen and allowed to solidify for 1 hour at 37°C. Approximately 10^3^ cells suspended in regular growth medium were seeded per well and protease activity was determined under a fluorescent microscope after 24 hours of incubation. Light and fluorescent pictures were collected under Olympus IX71 microscope with attached CCD camera. Fluorescent intensity measurement of collagen degradation was performed using the Olympus Microsuite software.

For protease expression and secretion studies, the same LRBM culture system, lacking the DQ collagen, was used. In addition, cells were serum starved overnight (fed with serum-free DMEM) before harvesting the conditioned medium. Corresponding total cell lysates were used for determining intracellular protein levels.

To determine the effect of SHP2 inhibition on differentiation of BTBC cells, the LRBM culture described previously [19,23] was used. Approximately 10^3^ cells were suspended in 250 μl of assay medium (DMEM supplemented with 2% horse serum, 10 μg/ml recombinant human insulin, 0.5 μg/ml hydrocortisone, 5 ng/ml EGF, 100 ng/ml cholera toxin and 1X penicillin/streptomycin) and seeded on a solidified LRBM and cultured at 37°C in 7% CO_2_ incubator. The cells were re-fed with assay medium every 4 days and phase contrast pictures were taken after 15 days.

### Hormone dependency and tamoxifen sensitivity

Cells were seeded in 96-well plates in DMEM containing 10% FBS plus or minus estradiol (E2) for 48 hours. The effect of E2 on cell growth was determined using a Luminescent cell viability assay (Promega) that measures growth based on ATP levels. The manufacturer’s protocol was followed in seeding cells, preparation of reagents, and luminescent measurements. For testing sensitivity to tamoxifen, cells were grown in the same way for 24 hours and then treated with vehicle (DMSO) or 5 μM tamoxifen for another 24 hours. Cell growth was measured in Synergen H3 (Biotech) plate reader and the data was analyzed with the IGEN-5 software.

## Results

### SHP2 is essential for the maintenance of the mesenchymal morphology in BTBC cells

**Figure 1:**
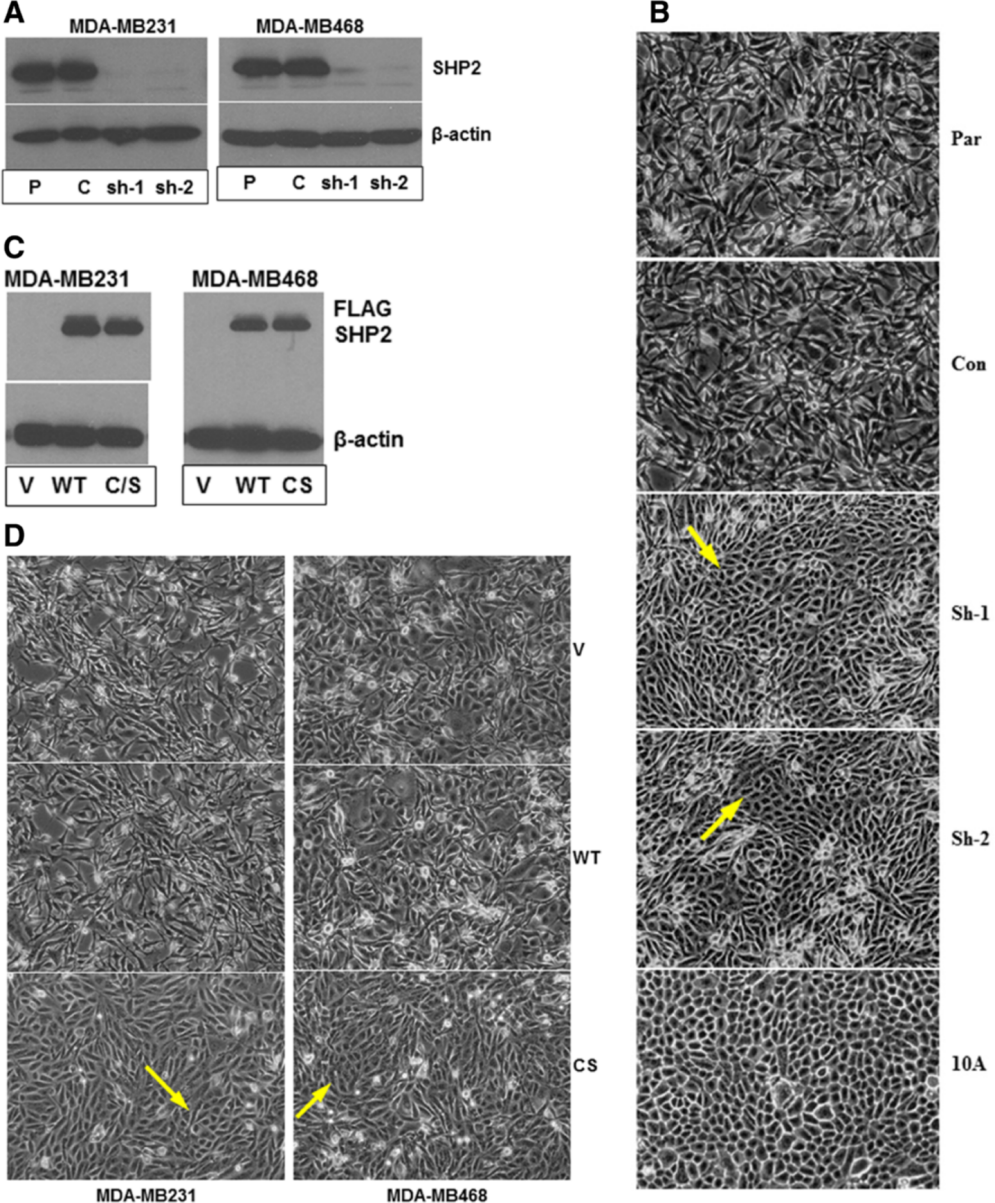
**Silencing SHP2 expression induces morphological changes in BTBC cells. A)** The expression of SHP2 was effectively silenced with two independent shRNA (shRNA-1 and shRNA-2) sequences. P: parental cells; C: control cells; sh-1: shRNA-1 cells; sh-2: shRNA-2 cells. **(B)** Pictures of parental, control and SHP2-silenced cells showing morphological changes induced by SHP2 silencing in the MDA-MB231 cells. A picture of the MCF-10A cells is included to show morphological similarity to the SHP2-silenced cells. **C)** Analysis of ectopic SHP2 expression; V: vector alone; WT: wild type SHP2; CS: C459S-SHP2. **D)** Pictures of MDA-MB231 and MDA-MB468 cells expressing vector alone, WT-SHP2 or C459S-SHP2 to show morphological changes. Note that expression of dominant-negative SHP2 (C459S-SHP2) induces morphological changes reminiscent of SHP2-silnced cells.

To determine the functional significance of SHP2 in BTBC, we chose the MDA-MB231 and the MDA-MB468 breast cancer cell lines which are known to be basal-like and triple-negative [24,25]. These cells harbor several genetic abnormalities commonly discovered in BTBC, including activating Ras mutation in the MDA-MB231, elevated EGFR expression and p53 mutation in both [24,26], and PTEN homo-deletion and EGFR gene amplification in the MDA-MB468 cells [24,27]. In addition, both cell lines show elevated SHP2 expression [28]. Hence, they are appropriate for testing the importance of SHP2 in BTBC. The expression of SHP2 was silenced with two independent shRNA sequences that were previously shown to be specific and devoid of off-target effects [18,21]. Cells expressing luciferase shRNA were used as controls in this study. As shown in Figure 1A, each shRNA effectively silenced SHP2 expression. Microscopic examination of confluent cells grown in 2D cultures revealed that the SHP2 silenced cells had acquired an epithelial morphology characteristic of luminal epithelial cells while the parental and the control cells showed the expected elongated and spindle-shaped mesenchymal morphology. In other words, silencing SHP2 expression induced morphological changes that are comparable to the MCF-10 cells, the immortalized and non-tumorigenic breast epithelial cells commonly used as “normal” controls (Figure 1B). Morphological changes induced by SHP2 silencing were obvious even in non-confluent cells (Additional file 1: Figure S1A). Similar results were obtained in the MDA-MB468 cells (Additional file 1: Figure S1B and C).

To further confirm that the PTPase activity of SHP2 was responsible for the observed morphological changes and to rule out the possibility of any off-target shRNA effects, we inhibited SHP2 function by expression of the PTPase-dead SHP2 (C459S-SHP2), also known as dominant-negative SHP2, using the strategy described by us previously [12,13]. As shown in Figure 1C, FLAG-tagged wild-type SHP2 (WT-SHP2) and C459S-SHP2 were efficiently expressed in both cells lines. In agreement with shRNA-based SHP2 inhibition, expression of C459S-SHP2 led to similar morphological changes, while the wild-type counterpart (WT-SHP2) or vector alone did not (Figure 1D). These results suggest that SHP2 is required for the maintenance of the mesenchymal property in BTBC cells and its inhibition induces luminal-like morphology in BTBC cells.

### SHP2 is essential for the migratory and invasive properties of BTBC cells

**Figure 2:**
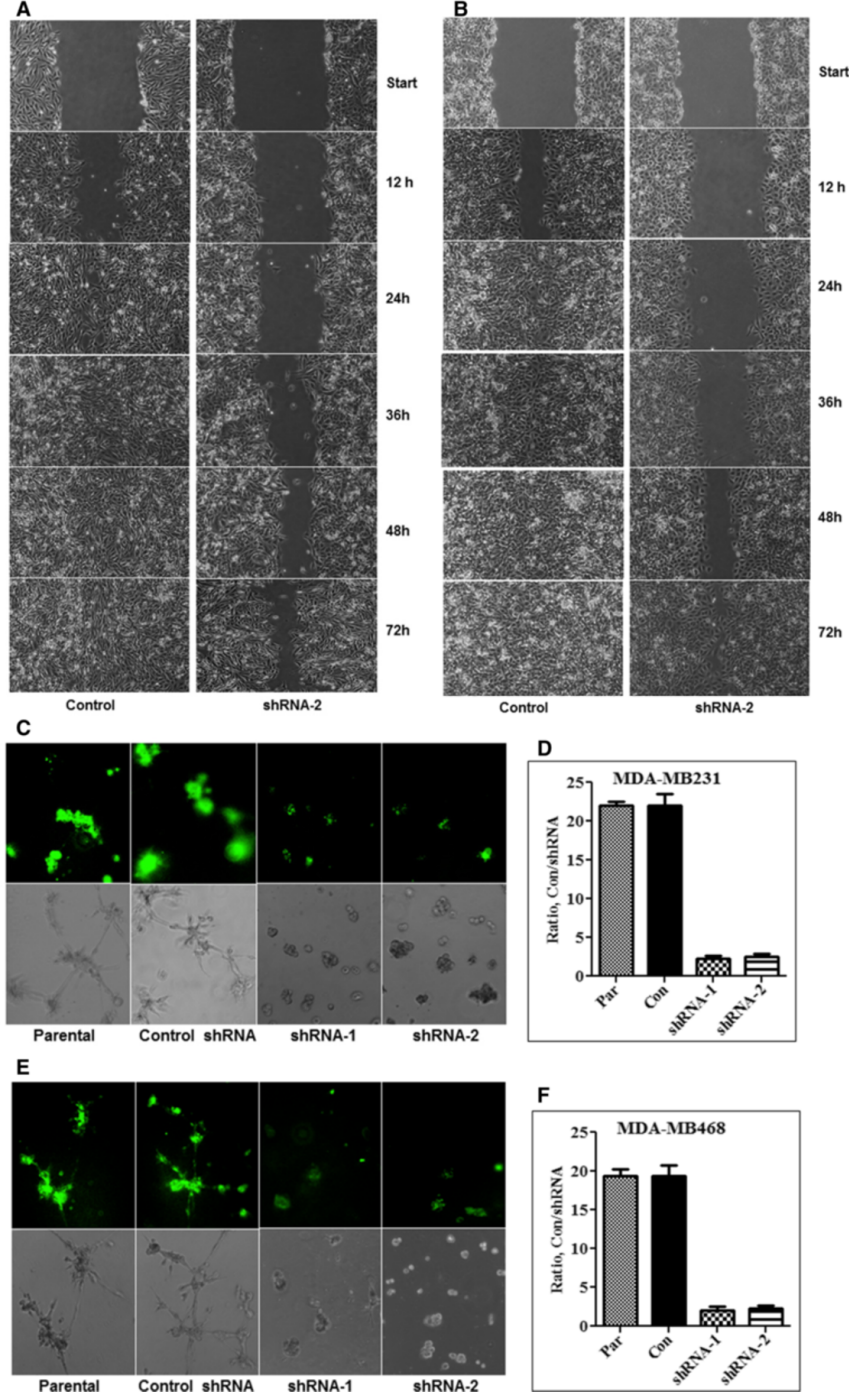
**Effect of SHP2 silencing on cell migration.** Silencing SHP2 expression retards cell migration in the MDA-MB231 **(A)** and the MDA-MB468 **(B)** cells. Data shown was from the control and the shRNA-2 cells derived from the respective cell lines. **C)** Effect of SHP2 silencing on the ability of the MDA-MB231 cells to degrade FITC-labeled collagen in 3D matrigel invasion assay. **D)** Fluorescent intensity measurement of collagen degradation by the MDA-MB231 cells. **E)** Effect of SHP2 silencing on the ability of the MDA-MB468 cells to degrade FITC-labeled collagen in 3D matrigel invasion assay. **F)** Fluorescent intensity measurement of collagen degradation by the MDA-MB468 cells. Data shown was mean ± SEM of three independent experiments.

Most BTBC cell lines, including the cells used in this study are highly mesenchymal and exhibit enhanced migratory and invasive properties [29]. The morphological changes induced by SHP2 inhibition were indicative of the importance of SHP2 in these properties. To test this possibility, we employed the simple wound-healing assay in which cells will be induced to migrate and fill the space created by scratching. In agreement with our previous report on effect of SHP2 in cell polarity [18], silencing SHP2 expression retarded cell migration. While the control cells were able to heal the wound in 24 hours, the SHP2-silenced cells were unable to do so even in 48 hours (Figure 2A and B). Similar results were obtained when SHP2 function was inhibited by expression of C459S-SHP2 (Additional file 2: Figure S2A and B). Hence, SHP2 is essential for the migratory behavior of BTBC cells.

The effect of SHP2 inhibition on the invasive property of BTBC cells was tested by the DQ matrigel assay. This assay system utilizes collagen IV conjugated to quenched-FITC, which fluoresces upon collagen degradation. Fluorescence emission is proportional to amount of collagen degradation, and therefore, it can be used to measure ability of cells to degrade and invade extracellular matrix (ECM). Cells were mixed with laminin-rich basement membrane (LRBM), containing approximately 10 μg/ml DQ collagen as described previously [22], and seeded in slide culture chambers. ECM degradation was monitored under a fluorescent microscope as described in the materials and methods. While the parental and the control cells showed enhanced matrigel degradation, the SHP2-silenced cells had very little or no degradation (Figure 2C and E). The corresponding light pictures further confirmed the highly mesenchymal and invading nature of the parental and control cells and the absence of such phenotypes in the SHP2-silened cells. Fluorescence intensity measurements further confirmed that the parental and the control cells degraded collagen matrices approximately 20 times more than the SHP2-silenced cells (Figure 2D and F). Expression of C459S-SHP2 also led to suppression of the invasive phenotype (Additional file 2: Figure S2C). These results clearly demonstrated that the invasive property of BTBC cells is SHP2 dependent.

### SHP2 is required for expression of EMT proteins and matrix-degrading enzymes

**Figure 3:**
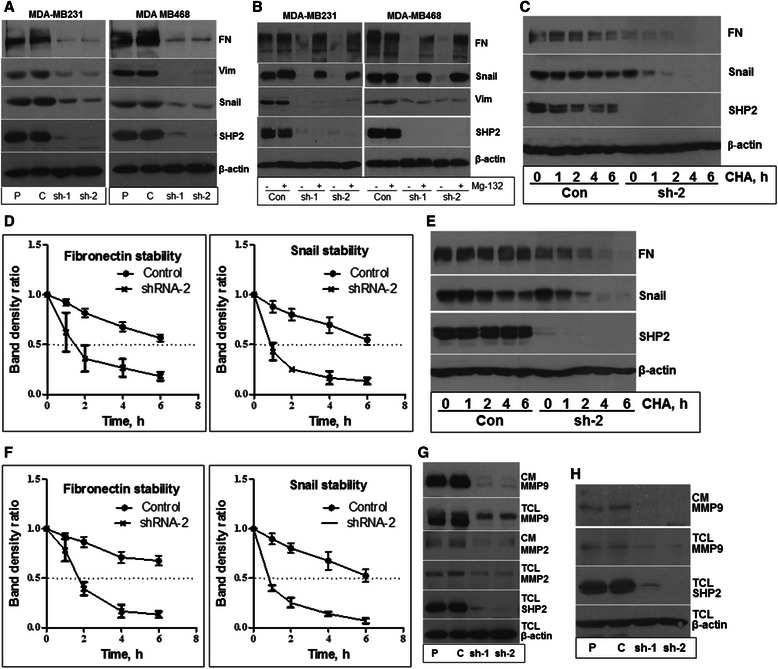
**Effect of SHP2 silencing on expression of EMT proteins. A)** Silencing SHP2 expression down regulated the levels of fibronectin (FN), Snail and vimentin (Vim). **B)** Proteasome inhibition with Mg-132 restores Snail and fibronectin levels, but not vimentin. **C)** Analysis of FN and Snail levels in the control and the SHP2-silenced MDA-MB231 cells after stabilization with Mg-132 and inhibition of new protein synthesis with cyclohexamide (CHA in a time-course (TC) fashion. **D)** Band density measurement of FN and Snail levels from three independent experiments performed as in **C**. **E)** Analysis of FN and Snail levels in the control and the SHP2-silenced MDA-MB468 cells. The experiments were performed as in **C**. **F)** Band density measurement of FN and Snail levels from three independent experiments performed as in **E**. **G)** Effect of SHP2 silencing on expression and secretion of MMP2 and MMP9 in MDA-MB231 cells. **H)** Effect of SHP2 silencing on expression and secretion of MMP9 in MDA-MB468 cells.

The retardation of cell migration and the blockade of invasiveness induced by SHP2 silencing implied loss of the mesenchymal phenotype. To further validate these observations, total cell lysates prepared from the 3D matrigel cultures were analyzed for expression of the mesenchymal markers, Snail, fibronectin (FN), and Vimentin. Consistent with the observed phenotypic changes, the levels of Snail, FN, and Vimentin were significantly reduced in the SHP2-silenced cells (Figure 3A), confirming that SHP2 is essential for the maintenance of the mesenchymal property in BTBC cells. Assuming that one of the mechanisms for SHP2 in promoting the expression of EMT proteins could be through protein stability, we conducted proteasome inhibition studies with Mg-132 treatment. As shown in Figure 3B, treating cells with Mg-132 restored FN and Snail, but not vimentin. These findings suggest that SHP2 promotes the stability of FN and Snail, but its positive effect on vimentin might be at the level of transcription.

The proteasome inhibition studies were suggestive of enhanced degradation of FN and Snail in the absence of SHP2. To examine the dynamics of FN and Snail degradation in the presence and absence of SHP2, we first restored these proteins by Mg-132 treatment for 6 hours. This was necessary since FN and Snail levels in the shRNA cells were low and hence could not be compared. After removal of Mg-132 by washing, new protein synthesis was blocked by cyclohexamide treatment for variable time points. As shown Figure 3C and E, the levels of both proteins decreased very rapidly in the SHP2-silenced cells, but very slowly in the controls. Band density measurements revealed that the half-life of FN was less than 2 hours while that of Snail was less than 1 hour in the SHP2 silenced cells. But, the half-life of both proteins in the control cells was at least 6 hours (Figure 3D and F). Hence, loss of SHP2 destabilizes some EMT proteins in BTBC cells.

Previous studies have shown that the MDA-MB231 and the MDA-MB468 cells express MMP2 and MMP9 [30,31]. We reasoned that the loss of the invasive property in the SHP2-silenced cells could be related to loss of the expression of these proteases. We thus tested the impact of SHP2 inhibition on the expression and secretion of MMP2 and MMP9 in matrigel assays. Cells were seeded in matrigel and the conditioned media harvested from these cultures was analyzed by immunoblotting for MMP-2 and MMP-9. Inhibition of SHP2 blocked the secretion of MMP9 into the medium in both the MDA-MB231 and the MDA-MB468 cells, although the level of MMP9 in the control MDA-MB468 cells was relatively low in the first place (Figure 3G and H, top panels). Analysis of corresponding total cell lysates showed that the expression of MMP9 was reduced, but not completely blocked by SHP2 inhibition in both cells (Figure 3G and H, second panels). However, the level of MMP9 in the control MDA-MB468 cells was relatively low when compared to the control MDA-MB231 cells, which also reflects the low level of MMP9 secretion into the medium by the MDA-MB468 cells. The secretion and expression of MMP2 was also suppressed in the SHP2-silenced MDA-MB231 cells (Figure 3G, third panel), but this protease was undetectable in the MDA-MB468 cells (data not shown). Reprobing for β-actin in the total cell lysates showed comparable level of protein loading. Overall, these results suggest that SHP2 promotes the invasive phenotype of BTBC cells by positively regulating the expression and secretion of MMP9 and possibly MMP2 in a cell context-dependent manner.

### Inhibition of SHP2 induces basal-to-luminal transition

**Figure 4:**
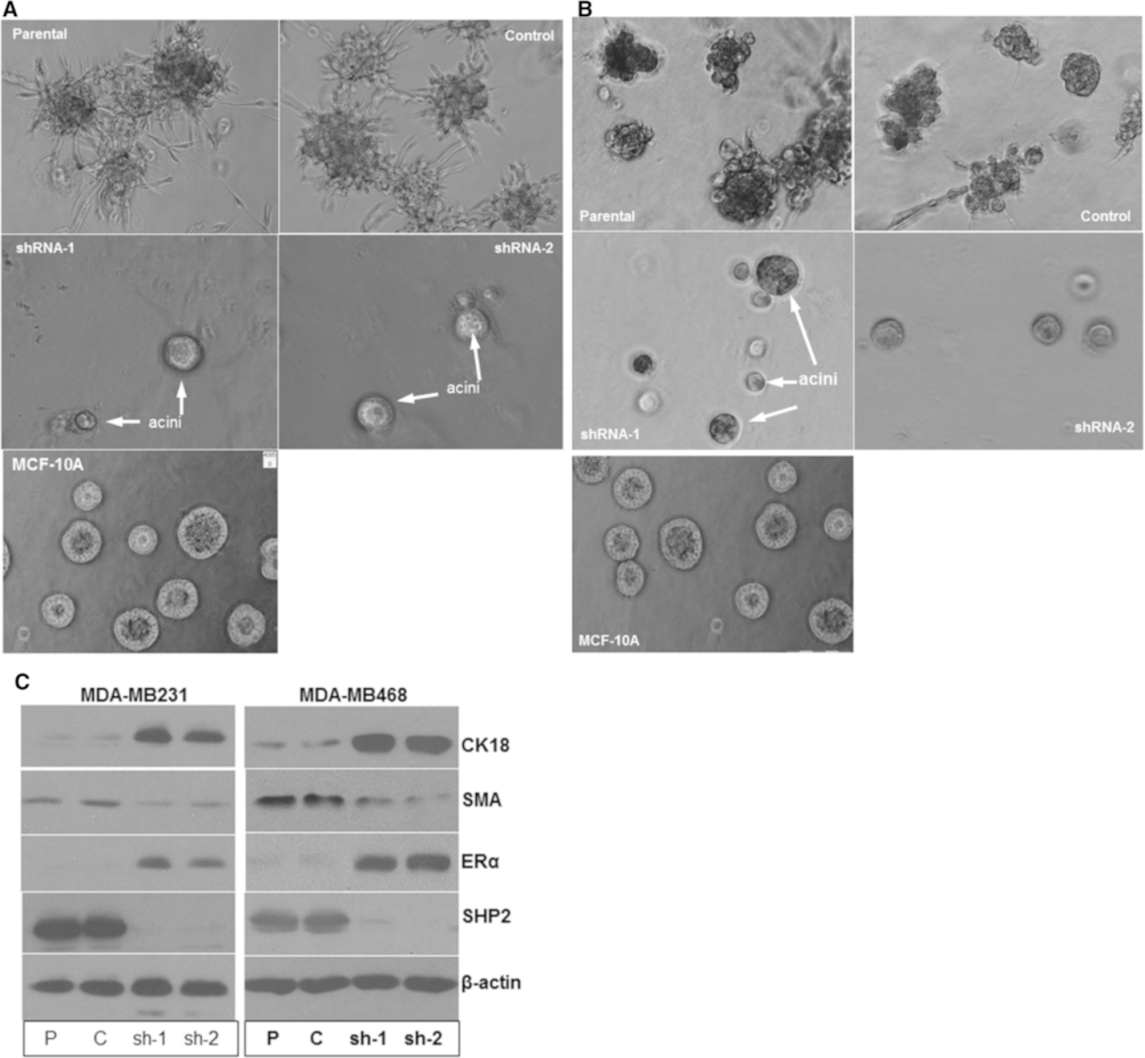
**Silencing SHP2 expression induces acini-like structure formation. A)** Light pictures of parental, control and SHP2-silenced MDA-MB231 cells cultured in 3D LRBM matrigel that allows acini-like structure formation. **B)** Light pictures of parental, control and SHP2-silenced MDA-MB468 cells cultured in 3D LRBM matrigel that allows acini-like structure formation. Pictures of acini-like structures formed by the MCF-10A cells cultured under identical conditions is shown in both **A** and **B** for comparison. **C)** Effect of SHP2 silencing on expression of basal and luminal markers and estrogen receptor alpha (ERα). Data presented is representative of at least three independent experiments.

As shown in Figure 1 and Additional file 1: Figure S1, silencing SHP2 expression induced a luminal-like epithelial morphology in BTBC cells. Based on these findings, we reasoned that inhibition of SHP2 may induce conversion of BTBC cells to a luminal lineage, which we termed basal-to-luminal transition (BLT). This possibility was tested by the laminin-rich basement membrane (LRBM) culture where non-transformed luminal breast epithelial cells like the MCF10A form acini-like structures while breast cancer cells produce a continuously-growing amorphous cellular mass [19,23]. As expected, the control cells formed a disorganized cellular mass that continued to grow and invade, whereas the SHP2-silenced cells grew very slowly, forming acini-like spheroid structures that are comparable to those formed by the control MCF-10A cells (Figure 4A and B). These results suggest that inhibition of SHP2 reverses the transformation and invasive phenotype and induces BLT in BTBC cells.

To further confirm the occurrence of BLT, the expressions of basal and luminal markers were analyzed by immunoblotting of total cell lysates prepared from LRBM cultures. The results showed that the expression of the basal marker α-SMA (alpha smooth muscle actin) was higher in the parental and the control cells and lower in the corresponding SHP2-silenced cells (Figure 4C). But, the converse was true for expression of the luminal marker CK18 (cytokeratin 18). These results confirm that inhibition of SHP2 leads to BLT in triple-negative breast cancer cells.

The next logical step was to determine whether induction of BLT is accompanied by loss of the triple-negative status. Normal luminal breast epithelial cells and breast cancer cells that belong to the luminal subtype express the hormone receptors estrogen receptor alpha (ERα) and progesterone receptor (PR). We thus determined the state of ERα and PR expression in the same total cell lysates. Consistent with the occurrence of BLT, inhibition of SHP2 led to expression of ERα in triple-negative breast cancer cells (Figure 4C). However, we were unable to detect PR expression by immunoblotting with a specific antibody (not shown). Nonetheless, these findings demonstrate that SHP2 is essential for the maintenance of the triple-negative status in BTBC cells. In other words, inhibition of SHP2 in BTBC cells induces differentiation to hormone-positive cells.

### Inhibition of SHP2 induces estrogen dependency and sensitivity to tamoxifen

**Figure 5:**
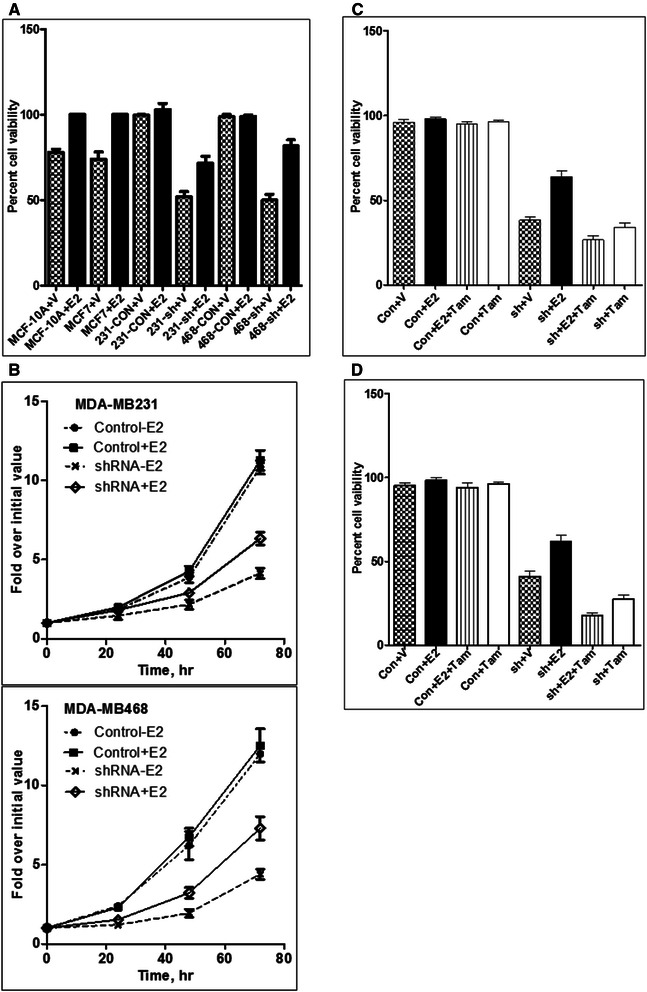
**Effect of SHP2 silencing on estrogen responsiveness. A)** Comparison of cells for responsiveness to estradiol treatment using cell viability assay. **B)** Effect of estradiol on the growth of the control and the SHP2-silenced MDA-MB231 and MDA-MB468 cells as determined by direct cell counting in a time-course fashion. **C)** Effect of SHP2 silencing on sensitivity to tamoxifen in the MDA-MB231 cells. **D)** Effect on SHP2 silencing on sensitivity to tamoxifen in the MDA-MB468 cells. V: vehicle; E2: estradiol; Tam: tamoxifen; Con: control shRNA; sh: SHP2 shRNA.

The expression of ERα (Figure 4C) in SHP2-silenced cells suggested that they may depend on estrogen signaling for growth. To test this possibility, we conducted cell growth studies in the presence and absence of estradiol (E2) using a cell viability assay (Promega) that determines cell growth based on ATP levels. The MCF-10A (non-transformed breast epithelial cells) and the MCF-7 (the hormone-positive breast cancer cell line) cells that require E2 for growth were used as positive controls. While the control cells did not respond to E2 treatment, the SHP2-silenced cells showed an approximately 30% increase in cell growth rate over the unstimulated counterparts (Figure 5A). As expected, the growth of the MCF-10A and the MCF-7 cells was retarded by E2 subtraction in the growth medium. We also determined the effect of E2 on cell growth rate by direct counting in a time-course fashion as described previously [32]. Consistent with the cell viability data, addition of E2 in the growth medium increased the growth rate of the SHP2-slinced cells. For instance, the unstimulated and the E2-stimulated SHP2-silenced cells grew by approximately 4-fold and 7-fold, respectively, over a period of 3 days (Figure 5B). As expected, the control cells grew by more than 10-fold over the same period of time. These observations suggest that SHP2 inhibition-induced ERα expression confers hormone dependency.

Based on the above findings, we reasoned that hormone dependency may lead to sensitivity to anti-hormone treatment. We used the same assay system to determine sensitivity to tamoxifen (Tam) by adding 1 μg/ml and incubation for 48 hours. While the control cells showed very little or no sensitivity, the SHP2 silenced cells exhibited loss of viability even below those that were not stimulated with E2 (Figure 5C and D). In other words, Tam abrogated the effect of E2 on cell growth in the SHP2-silenced cells. However, treating SHP2-silenced cells with Tam alone did not induce further inhibition than that induced by SHP2 silencing. These findings further support our hypothesis that inhibition of SHP2 induces ERα expression, hormone dependency, and sensitivity to anti-hormone therapy.

## Discussion

When compared to the hormone- and HER2-positive subtypes of breast cancer, BTBC causes disproportionately high mortalities in women [2,33,34]. The major factors are the aggressive nature of the disease and the lack of targeted therapies. Several RTKs, including EGFR, FGFR, c-MET, IGF-1R, and Eph2 are known to be dysregulated in BTBC [5,6,8], but development of targeted therapies against them has not been successful. SHP2 is a critical downstream signal transducer of many RTKs, including those dysregulated in BTBC [9-11,13,35,36], but its functional role has not been effectively demonstrated. As a result, its potential for targeted therapy in BTBC has not been explored. In this study, we conducted shRNA-based inhibition studies to show the importance of SHP2 in BTBC.

Most BTBC cell lines, including the cells used in this study are highly mesenchymal and exhibit enhanced migratory and invasive properties [29]. We found that silencing SHP2 expression induces morphological changes that resemble luminal breast epithelial cells which are apparent even in non-confluent cells (Figure 1B and Additional file 1: Figure S1). Expression of the PTPase-dead (dominant-negative) SHP2 also induced similar phenotypes (Figure 1D), confirming that SHP2 is essential for the mesenchymal morphology of BTBC cells. These findings are consistent with our previous report in which we showed the importance of SHP2 for the maintenance of the transformed phenotype in HER2-positive and basal-like breast cancer cells [19]. Therefore, SHP2 may play critical roles in different subtypes of breast cancer.

We have recently demonstrated that SHP2 regulates focal adhesion kinase (FAK) in BTBC cells to promote cell polarity and migration in 2D cultures [18]. Because the SHP2 inhibition-induced loss of the mesenchymal morphology was so dramatic, we wanted to determine the effect of SHP2 silencing on cell migration for extended period of time. We have found that the SHP2 silenced cells could not completely close the monolayer wound even in 48 hours, while the control cell did it within 24 hours (Figure 2A and B). Expression of dominant-negative SHP2 also led to retardation in cell migration (Additional file 2: Figure S2A and B), confirming the importance of the SHP2 enzyme function in cell migration. Thus, inhibition of SHP2 severely retards BTBC cell migration.

In agreement with the loss of the mesenchymal morphology and migratory behavior in 2D, the SHP2-silenced cells were defective in their invasive property, the hallmark of “mesenchymalness” (Figure 2C-F and Additional file 2: Figure S2C). These changes were confirmed by the reduced expression of Vimentin, FN, and Snail, the commonly used EMT markers (Figure 3A). Interestingly, proteasome inhibition restored Snail and FN expression, but not vimentin (Figure 3B). Time-course protein translation inhibition studies following Mg-132 stabilization showed that loss of SHP2 leads to rapid degradation of FN and Snail by the proteasome (Figure 3C-F). These findings suggest that SHP2 promotes FN and Snail protein stability in BTBC cells. However, our data does not show the mechanism by which SHP2 promotes the stability of FN and Snail. Future studies addressing aspect will be necessary. In case of vimentin, it seems that SHP2 promotes vimentin expression at the transcriptional level. It will also be necessary to address this point in future studies.

Ability to interact, degrade, and move through the ECM by a cancer cell is the major mechanism for the development of a malignant disease [37,38]. Matrix metalloproteinase 2 and 9 (MMP-2 and MMP-9) have been implicated as playing significant roles in enabling invasiveness and metastasis of breast cancer [31,39]. We have demonstrated that inhibition of SHP2 suppresses the expression of both proteases in BTBC cells (Figure 3G and H). In support of our findings, a previous study has also shown that SHP2 promotes the expression of MMP9 [40]. These results provide a mechanistic explanation as to how SHP2 promotes the invasiveness property of BTBC cells. However, our results cannot rule out the possibility of SHP2 regulating the expression and/or secretion of additional proteases to promote invasiveness. Future studies may also be needed to explore these possibilities.

For the first time, we have demonstrated that inhibition of SHP2 induces basal-to-luminal transition (BLT) in BTBC cells (Figure 4A and B). Previous studies by us and others have shown that the acini-like structures formed by isolated breast epithelial cells resemble the milk-producing structures of the normal breast. Therefore, our results are consistent with SHP2 inhibition leading to BLT. More surprising was the induction of ERα expression (Figure 4C), which suggested that inhibition of SHP2 converts BTBC cells into hormone-positive epithelial cells.

The SHP2-inhibition induced BLT in BTBC cells was unexpected since these cells are thought to originate from the basal cells of the breast, the myoepithelial cells. One possible explanation is that the minority stem-like cell populations, also known as cancer stem cells (CSC), present in the MDA-MB-231 and the MDA-MB-468 cells [41,42], may give rise to luminal lineage cells. As in normal breast stem cells which are pluripotent and can give rise to luminal and basal lineages, the stem-like cells present in BTBC cells might have differentiated into luminal lineage upon SHP2 inhibition, leading to formation of acini-like structures in LRBM cultures. Future studies may be needed to show how inhibition of SHP2 in BTBC cells leads to differentiation of pluripotent basal cells to luminal cells.

We have demonstrated that inhibition of SHP2 converts triple-negative breast cancer cells into hormone-positive (ERα-positive) cells that depend on estrogen signaling for growth (Figure 5A and B). We have also shown that SHP2-silenced cells are sensitive to tamoxifen treatment (Figure 5C and D). A recent study has shown that SHP2 is essential for estradiol (E2)-induced cell growth and signaling in the ERα-positive MCF7 breast cancer cell line primarily using a small molecule-based SHP2 inhibition [43]. This report also showed cross-talk between the ERα and the IGF-1R-Gab2 signaling axis in which SHP2 was found to be important. It is possible that the observed inhibition of cell growth and signaling in MCF7 cells treated with the SHP2 inhibitor PHPS1 might have emanated from the loss of this cross talk. Since activation of the SHP2 enzyme function is dependent on tyrosine kinase activation and interaction of its SH2 domains, it is unlikely that ERα per se can activate SHP2 to mediate signaling. In addition, the observed differences between our findings and this report may reflect cell-context differences between the MCF7 and the SHP2-silnced BTBC cells. Nonetheless, our findings imply that SHP2 inhibition can be used to treat BTBC singly or in combination with the existing anti-hormone therapies.

## Conclusions

In this study, we show that inhibition of SHP2 induces basal-to-luminal transition (BLT), ERα expression, estrogen dependency for growth, and sensitivity to anti-hormone therapy. Therefore, inhibition of SHP2 may provide a therapeutic benefit to basal-like and triple-negative breast cancer patients if specific drugs can be developed.

## Additional files

Additional file 1: Figure S1. Includes pictures of non-confluent parental, control, and SHP2 shRNA cells derived from the MDA-MB231 (A) and from the MDA-MB468 (C). Also shown in Figure S1 are pictures of confluent parental, control, and SHP2 shRNA cells derived from the MDA-MB468 cells (B). Pictures of non-confluent parental MCF-10A cells are included in all cases as a reference.
Additional file 2: Figure S2. In this supplemental data, we show that inhibition of SHP2 by dominant-negative (C459S-SHP2) expression also suppresses cell migration and matrigel invasion.

## Abbreviations

BTBC: basal-like and triple-negative breast cancer
RTK: receptor tyrosine kinase
EGFR: epidermal growth factor receptor
SHP2: Src homology 2 phosphotyrosyl phosphatase
ERα: estrogen receptor alpha
the MET receptor: also known as hepatocyte growth factor receptor
EMT: epithelial-t-mesenchymal transition
MET: mesenchymal to epithelial transition
BLT: basal-to-luminal transition
LRBM: laminin-rich basement membrane
