# Supplementary material for: Inhibition of SHP2 in basal-like and triple-negative breast cells induces basal-to-luminal transition, hormone dependency, and sensitivity to anti-hormone treatment
Source: BMC Cancer. 2015 Mar 8;15:109. doi: 10.1186/s12885-015-1131-2 (PMC4359540; doi:10.1186/s12885-015-1131-2)
Supplement: Additional file 1: Figure S1. — Includes pictures of non-confluent parental, control, and SHP2 shRNA cells derived from the MDA-MB231 (A) and from the MDA-MB468 (C). Also shown in Figure S1 are pictures of confluent parental, control, and SHP2 shRNA cells derived from the MDA-MB468 cells (B). Pictures of non-confluent parental MCF-10A cells are included in all cases as a reference. [file 12885_2015_1131_MOESM1_ESM.docx]

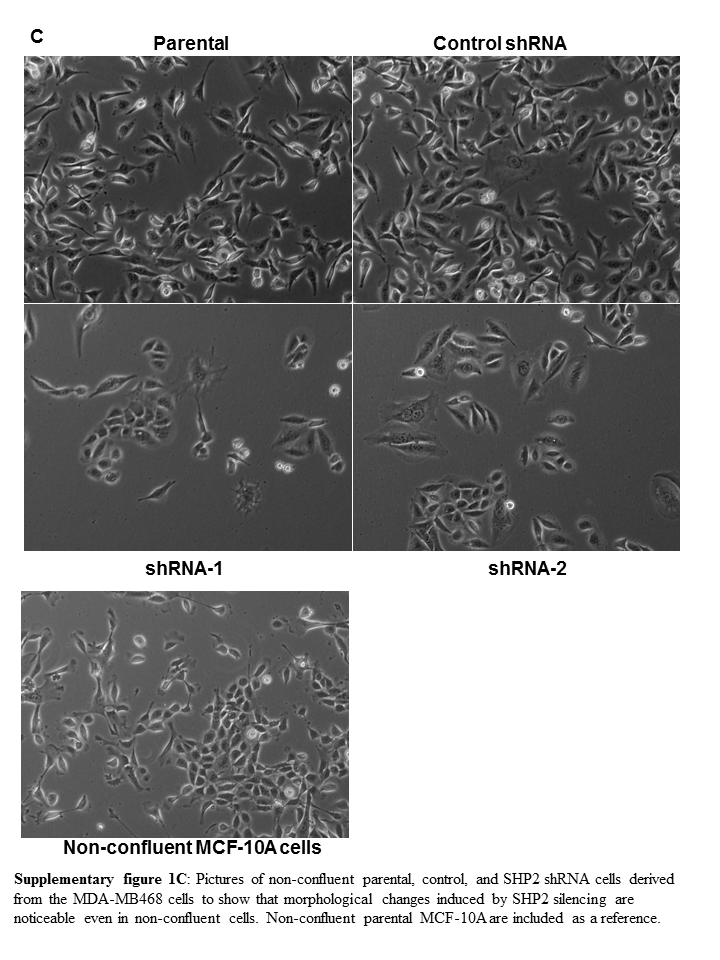

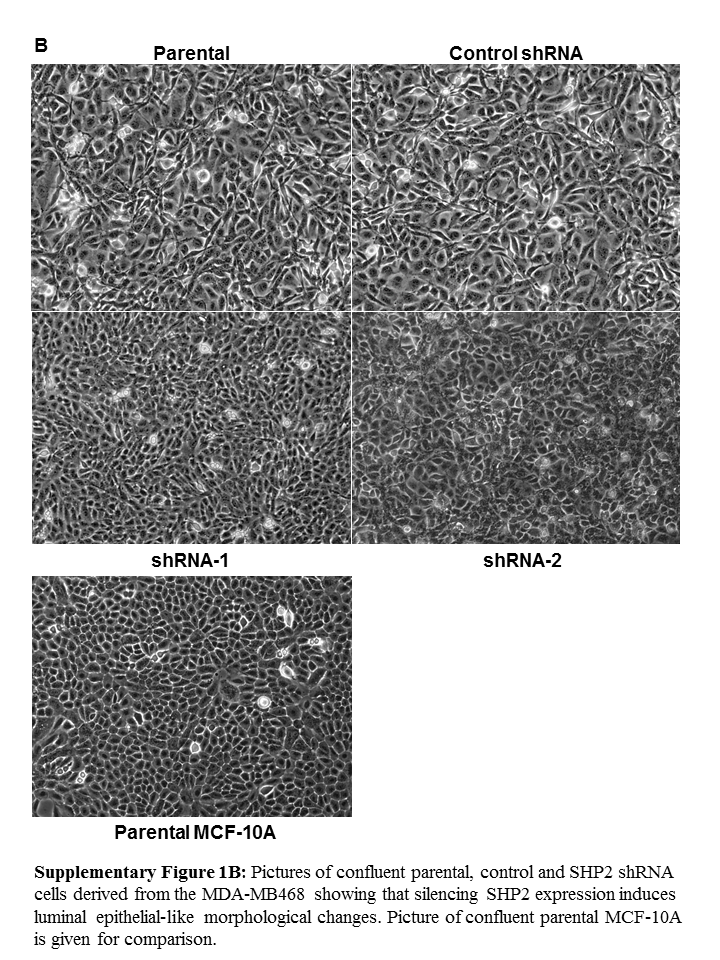

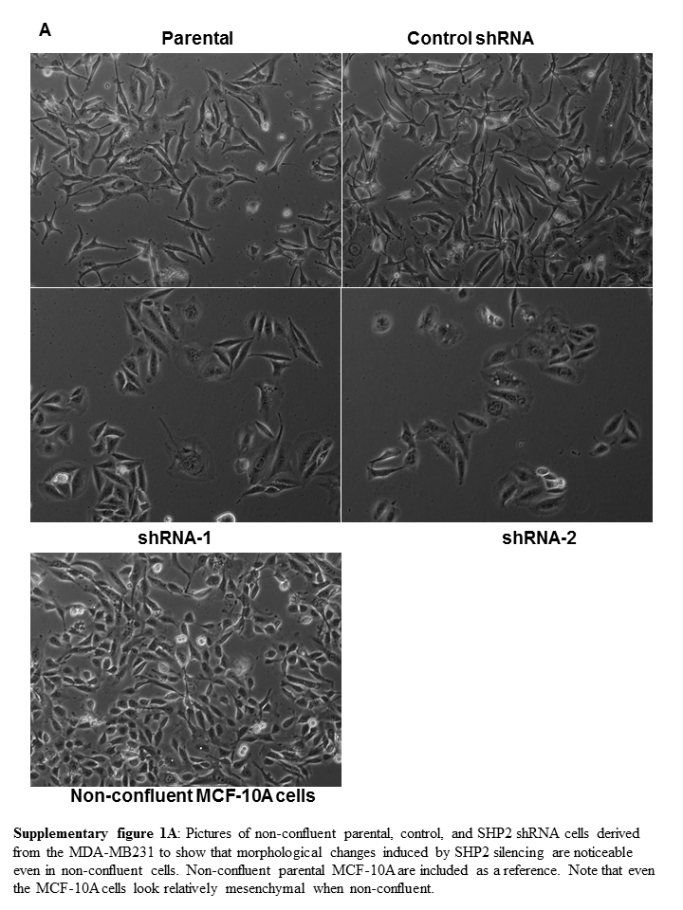


**Supplementary figure 1: A**) Pictures of non-confluent parental, control, and SHP2 shRNA cells derived from the MDA-MB231. B) Pictures of confluent parental, control, and SHP2 shRNA cells derived from the MDA-MB468 to show that SHP2 silencing induces similar morphological changes in these cells as well. C) Pictures of non-confluent parental, control, and SHP2 shRNA cells derived from the MDA-MB468. The non-confluent pictures in both cell lines show that morphological changes induced by SHP2 silencing are noticeable even in non-confluent cells. Non-confluent parental MCF-10A are included in both cell line pictures as a reference. Note that even the MCF-10A cells look relatively mesenchymal when non-confluent.
