# Supplementary material for: Inhibition of SHP2 in basal-like and triple-negative breast cells induces basal-to-luminal transition, hormone dependency, and sensitivity to anti-hormone treatment
Source: BMC Cancer. 2015 Mar 8;15:109. doi: 10.1186/s12885-015-1131-2 (PMC4359540; doi:10.1186/s12885-015-1131-2)
Supplement: Additional file 2: Figure S2. — In this supplemental data, we show that inhibition of SHP2 by dominant-negative (C459S-SHP2) expression also suppresses cell migration and matrigel invasion. [file 12885_2015_1131_MOESM2_ESM.docx]

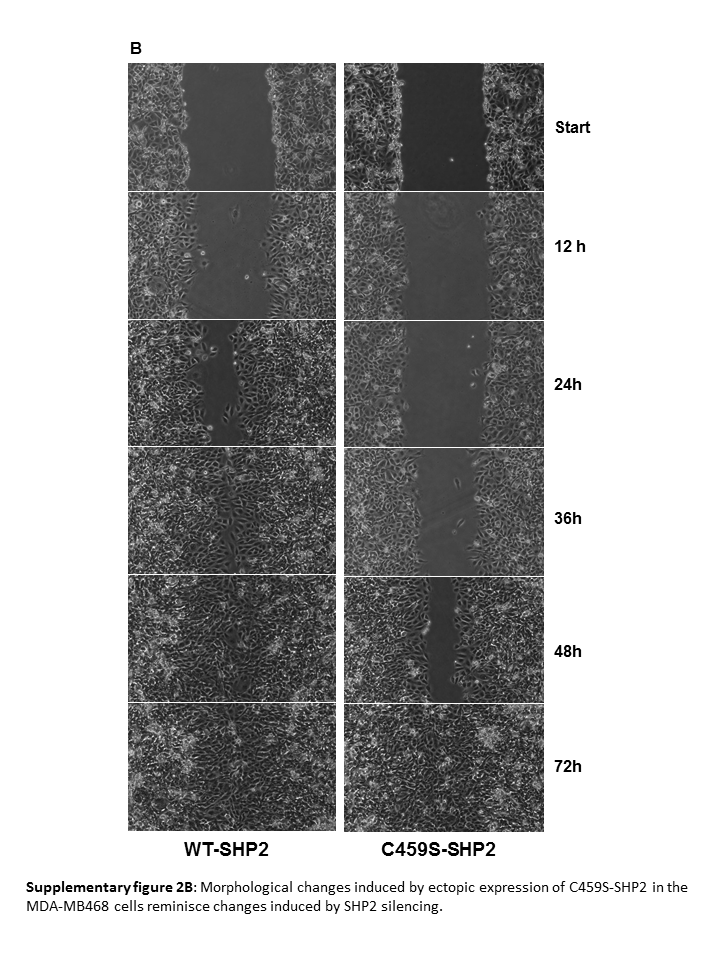

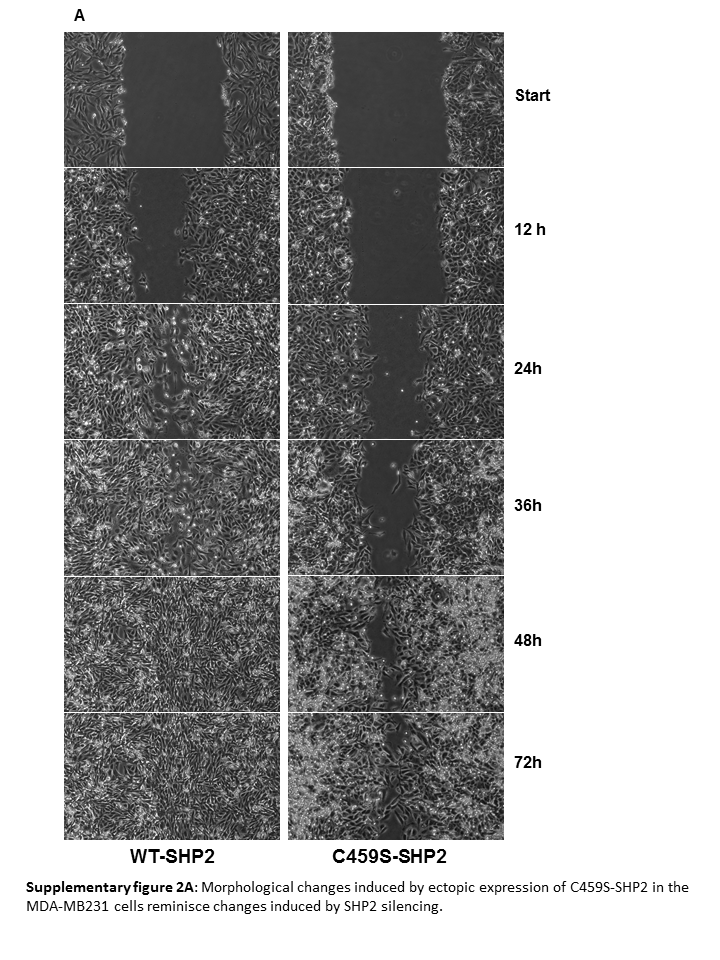

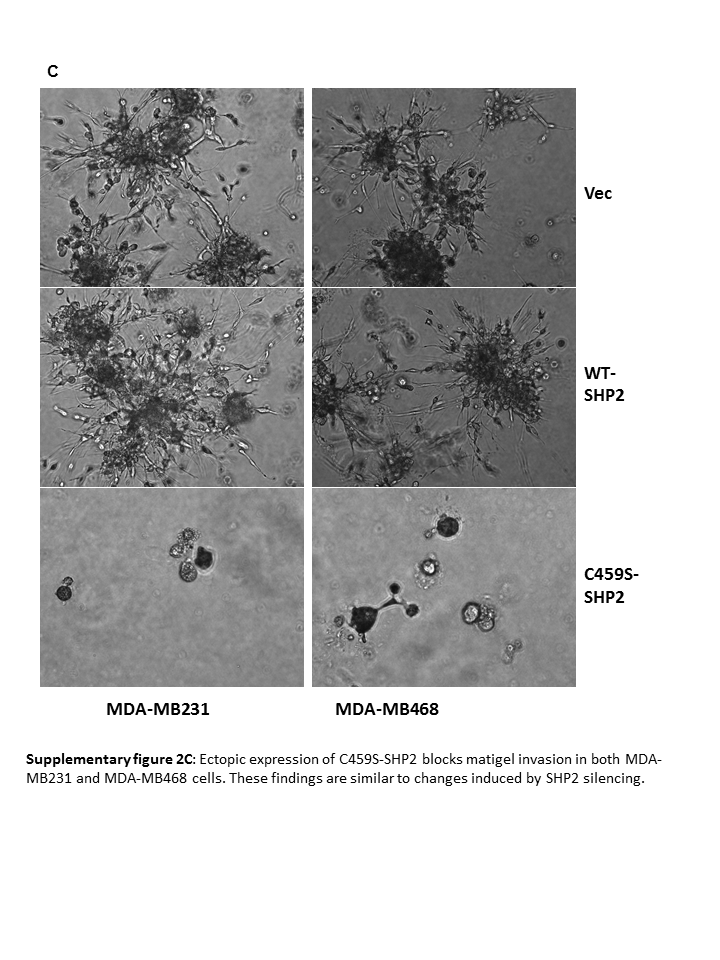


**Supplementary figure 2: A)** Inhibition of SHP2 by dominant-negative (C459S-SHP2) expression in the MDA-MB231 cells suppresses cell migration. B) Inhibition of SHP2 by dominant-negative (C459S-SHP2) expression in the MDA-MB468 cells suppresses cell migration. C) Inhibition of SHP2 by dominant-negative (C459S-SHP2) expression in the MDA-MB231 cells suppresses matrigel invasion by both the MDA-MB231 and the MDA-MB468 cells. But, expression of the wild-type counterpart (WT-SHP2) did not affect these phenotypes.
